# Relationship between histopathological findings and RAI in non-papillary follicular cell–derived thyroid carcinoma

**DOI:** 10.1210/jendso/bvag120

**Published:** 2026-05-21

**Authors:** Haruhiko Yamazaki, Kiminori Sugino, Ryohei Katoh, Miho Kawaida, Kenichi Matsuzu, Wataru Kitagawa, Aya Saito, Koichi Ito

**Affiliations:** Department of Breast and Thyroid Surgery, Yokohama City University Medical Center, Yokohama City, Kanagawa 232-0024, Japan; Department of Surgery, Ito Hospital, Tokyo 150-8308, Japan; Department of Surgery, Ito Hospital, Tokyo 150-8308, Japan; Department of Pathology, Ito Hospital, Tokyo 150-8308, Japan; Department of Pathology, Ito Hospital, Tokyo 150-8308, Japan; Department of Surgery, Ito Hospital, Tokyo 150-8308, Japan; Department of Surgery, Ito Hospital, Tokyo 150-8308, Japan; Department of Surgery, Yokohama City University School of Medicine, Yokohama City, Kanagawa 236-0004, Japan; Department of Surgery, Ito Hospital, Tokyo 150-8308, Japan

**Keywords:** differentiated high-grade thyroid carcinoma, follicular thyroid carcinoma, poorly differentiated thyroid carcinoma, radioactive iodine treatment

## Abstract

**Context:**

Detailed studies considering the distinct types of non-papillary follicular cell–derived thyroid carcinoma in terms of radioactive iodine (RAI) avidity are limited.

**Objective:**

This study aimed to investigate clinicopathological characteristics, relationship between histopathological findings, including solid/trabecular/insular (STI) components and RAI treatment (RAIT), in patients with non-papillary follicular cell–derived thyroid carcinoma.

**Methods:**

This study included 114 patients with non-papillary follicular cell–derived thyroid carcinoma who underwent first RAIT between January 2005 and August 2025.

**Results:**

Of the 114 patients, 5 (4%) were minimally invasive follicular thyroid carcinoma (MI-FTC), 41 (36%) were encapsulated angio-invasive FTC (EA-FTC), 23 (20%) were widely invasive FTC (WI-FTC), 22 (19%) were differentiated high-grade thyroid carcinoma (DHGTC), 21 (18%) were poorly differentiated thyroid carcinoma (PDTC), and 2 (2%) were others, respectively. The median percentages of STI component was 40% (range, 0%-10%) in all patients, 5% (range, 0%-50%) in MI-FTC, 30% (range, 0%-100%) in EA-FTC, 30% (range, 0%-100%) in WI-FTC, 28% (range, 5%-70%) in DHGTC, and 70% (range, 50%-100%) in PDTC, respectively. Among the 114 patients, the rate of RAI avidity was 60% (95% CI, 49%-71%) (47/78) in lung, 89% (95% CI, 79%-96%) (59/66) was in bone, 100% (13/13) in lymph node, and 100% (3/3) in other sites, respectively.

**Conclusion:**

RAI avidity was relatively high in non-papillary follicular cell–derived thyroid carcinoma, regardless of the histological type and percentage of STI components.

Follicular thyroid carcinoma (FTC) is the second most common histological type of thyroid carcinoma [1]. The fifth edition of the World Health Organization (WHO) classification divides FTCs into the following 3 categories based on their degree of invasiveness and vascular invasion: minimally invasive follicular thyroid carcinoma (MI-FTC), encapsulated angio-invasive follicular thyroid carcinoma (EA-FTC), and widely invasive follicular thyroid carcinoma (WI-FTC) [2]. FTC is more likely to metastasize to distant organs than to regional lymph nodes because of its tendency to invade blood vessels, and bone metastasis occurs more frequently than in papillary thyroid carcinoma (PTC) [3]. Poorly differentiated thyroid carcinoma (PDTC) is a rare tumor that originates from the thyroid follicular cells and displays predominant solid/trabecular/insular (STI) growth patterns, which may coexist with more or less extensively differentiated components of the follicular or papillary type on one side, and anaplastic carcinoma type on the other [4, 5]. PDTC often have follicular structural components, and the frequency of *RAS* mutations is higher than that of *BRAF* mutations, suggesting that poorly differentiated carcinomas are often derived from follicular tumors [6, 7].

Radioactive iodine treatment (RAIT) is a first-line systemic treatment commonly used to manage differentiated thyroid carcinoma (DTC) with distant metastasis. This approach significantly enhances survival and disease control, especially in patients with RAI avidity [8]. Several factors have been associated with lower RAI avidity in distant metastatic sites, such as patient age, large tumor size, histological type, [18F]-fluorodeoxyglucose positivity, and genetic mutations [9-13]. The recommendations for the use of RAIT in distant metastatic DTC are mostly based on experience with PTC, and the number of detailed studies considering the differences among the different types of DTC in terms of RAI avidity and response are limited. Additionally, RAI avidity may differ depending on pathological morphology, even within the same histological type [10]. However, the association between RAI avidity and distinct types of non-papillary follicular cell–derived thyroid carcinoma has not been fully investigated. It is important that early prediction of RAI-refractory patients helps avoid unnecessary RAIT and does not delay potentially more effective treatments, such as redifferentiation therapy [14] or systemic drug therapy [15].

This study aimed to investigate clinicopathological characteristics, relationship between histopathological findings including percentage of STI components and first RAIT, and predictive factors for RAI avidity of distant metastatic sites in patients with non-papillary follicular cell–derived thyroid carcinoma treated with RAI at our hospital. We also investigated the clinical outcomes of these patients.

## Materials and methods

### Study participants

The study protocol was approved by the Ethics Review Board of Ito Hospital (approval no. 472). This study was performed in accordance with the 1964 Declaration of Helsinki and its later amendments. We performed a retrospective analysis of 551 patients with follicular cell–derived thyroid carcinoma who underwent RAIT between January 2005 and August 2025 at Ito Hospital. After excluding patients with PTC, and patients treated with second or more RAIT, we identified 151 patients with non-papillary follicular cell–derived thyroid carcinoma. Among these 151 patients, a primary thyroid tumor specimen was not available in 37 patients. Ultimately, the study included population consisted of 114 patients after reviewing by 2 pathologists with a special interest in thyroid neoplasia (R.K. and M.K.), based on the fifth edition of the WHO classification [2]: 5 (4%) MI-FTC, 41 (36%) EA-FTC, 23 (20%) WI-FTC, 22 (19%) DHGTC, 21 (18%) PDTC, and 2 (2%) others. This study cohort included some FTC patients who were included in our previous study [16]. Among the 114 patients, 57 (50%) were diagnosed with thyroid carcinoma before 2013. Molecular data was not available in this cohort because molecular testing is not routinely performed before RAIT in Japan.

### Pathological analysis

A histopathologic review of slides obtained for clinical practice was conducted, with the pathologist blinded to the patients’ clinical characteristics or outcomes. Pathological analysis was re-reviewed for this paper in all 114 primary thyroid tumors. Thyroid carcinoma was classified according to the fifth edition of the WHO criteria [2]. Differentiated high-grade thyroid carcinoma (DHGTC) is an invasive high-grade follicular cell–derived carcinoma that is still differentiated because it retains the distinctive architectural and/or cytologic properties of well-differentiated histological types of carcinomas of follicular cell derivation, such as nuclear features and/or architecture of PTC and FTC [2]. DHGTC was defined as a tumor with a mitotic count ≥5/2 mm^2^ and/or tumor necrosis [2]. DHGTC and PDTC are high-grade non-anaplastic follicular cell–derived carcinomas (HGFCTCs) [2]. The TNM Staging System classification of all cases was performed in accordance with the criteria described in the Union for International Cancer Control/American Joint Committee Cancer 8th edition [17]. Patients who had distant metastasis at the diagnosis were defined as M1, and who developed distant metastasis during the follow-up period were defined as M2. The mechanism of action of RAIT includes some steps, such as selective uptake by thyroid cells and RAI accumulation, which may be associated with normal follicular structure of the thyroid gland [10]. Therefore, the percentage of STI components was also assessed. Although most cases included the entire tumor in the specimen, in older cases where complete section sets were unavailable, the STI components were assessed on the largest cut surface of the tumor.

### RAI treatment protocols and laboratory data

All 114 patients in the ablation group were instructed to follow a low-iodine diet for 2 weeks before postoperative ablation. To attain an increased thyroid-stimulating hormone level, all patients discontinued thyroxine 4 weeks before the administration of I-131 and thereby underwent thyroid hormone withdrawal. Three days after the administration of I-131, whole-body I-131 scintigraphy (WBS) was performed, and thyroglobulin (Tg) levels were determined. Between October 2004 and February 2015, Tg was quantified by an electrochemiluminescence immunoassay (ECLusys Tg, Roche Diagnostics) with functional sensitivity 0.5 ng/mL; from March 2015, an assay with functional sensitivity of 0.1 mg/mL was used (ECLusys TgII, Roche Diagnostics). TgAb positivity was defined as serum TgAb ≥ 40 IU/mL (ECLusys Anti-Tg, Roche Diagnostics), which interfered with Tg measurement.

### Definitions

After post-RAIT WBS, RAI avidity was assessed in the individual metastatic disease sites. If the individual known metastatic disease sites showed RAI accumulation, they were considered as having RAI avidity. If the metastatic disease sites or the largest and most clinically significant part did not show evidence of RAI accumulation, they were considered as having RAI non-avidity [10]. Radiology reports created by board-certified nuclear medicine physicians using post-therapy WBS for the sites of RAI accumulation were reviewed to determine RAI avidity. Image examinations including computed tomography or magnetic resonance imaging were used for identifying metastatic diseases. Mixed cases where both presence and absence of RAI accumulation were observed in same organ were considered as having RAI avidity. Equivocal cases such as extremely faint RAI accumulation were considered as having RAI non-avidity.

Tg levels were evaluated 6 months after RAIT to determine whether RAIT was effective. A Tg level reduction of ≥50% was considered significant for clinical usefulness of RAIT [18].

RAI-refractory disease was defined according to the 2015 American Thyroid Association guidelines [19]: (i) locoregional or metastatic disease that never concentrates RAI; (ii) tumor tissue that loses its ability to concentrate RAI; (iii) RAI is concentrated in some of the lesions, but not others; and (iv) metastatic disease that progresses despite the concentration of RAI.

Cutoff values of 40% for percentage of STI components, 1800 ng/mL for Tg level at the diagnosis, 80 ng/mL for Tg level before the first RAIT, and 500 ng/mL for stimulated Tg level at the first RAIT were used for subsequent analyses. Those values were obtained from median values of baseline characteristics.

For patients diagnosed with thyroid carcinoma via biopsy of distant metastasis, the diagnostic point was defined as the date on which the histopathology report confirmed thyroid carcinoma. For the remaining patients, the diagnostic point was defined as the date on which histopathology following initial thyroid surgery confirmed thyroid carcinoma.

Cause-specific survival from the diagnosis (CSS-D) was calculated as the interval from diagnosis to death due to thyroid carcinoma, while cause-specific survival from the first RAIT (CSS-R) was calculated as the interval from the first RAIT to death due to thyroid carcinoma. The median follow-up duration for the censored cases was 8.9 years.

### Statistical analysis

All statistical analyses were conducted using EZR (Saitama Medical Center, Jichi Medical University, Saitama, Japan), a graphical user interface for R (R Foundation for Statistical Computing, Vienna, Austria). EZR is a modified version of the R commander designed to add statistical functions frequently used in biostatistics [20]. Between-group differences in patient characteristics were tested for statistical significance. Categorical variables were compared using Fisher's exact test or the chi-square test. Continuous variables were compared using the Mann–Whitney U test. CSS-D and CSS-R were calculated using the Kaplan-Meier method and log-rank test. Statistical significance was set at *P* < .05. For the survival analysis, the final examination date was confirmed using the medical records of the study participants.

## Results

### Baseline patient characteristics

The baseline patient characteristics are shown in Table 1. Of the 114 patients, 28 (25%) were male and 86 (75%) were female. The median age was 59 years at diagnosis (range, 10-77) and 61 years at the first RAIT (range, 11-78). The median tumor size was 46 mm (range, 10-123), and the median preoperative Tg level was 1747.0 ng/mL (range, 11.0-81,644.0). A cutoff value of 1800 ng/mL for Tg level at the diagnosis was used for subsequent analyses. Seventy (61%) patients had distant metastasis at the diagnosis (M1) and the remaining 44 (39%) developed distant metastasis during the follow-up period (M2). The frequencies of disease sites at the first RAIT were as follows: lung, n = 78 (68%); bone, n = 66 (58%); lymph node, n = 13 (11%); and other sites, n = 3 (3%). Seventy-one patients had distant metastasis in a single organ (lung, n = 36; bone, n = 28; lymph node, n = 7). The remaining 43 patients had distant metastases in 2 or more organs (lung and bone, n = 35; lung and lymph node, n = 4; lung and other, n = 1; lung, bone, and other, n = 2; bone and lymph node, n = 1). Thirty-three patients (29%) underwent systemic drug therapy for RAI-refractory progressive disease. The histological classifications were as follows: MI-FTC, n = 5 (4%); EA-FTC, n = 41 (36%); WI-FTC, n = 23 (20%); DHGTC, n = 22 (19%; diagnosed as MI-FTC in 1, EA-FTC in 13, and WI-FTC in 8 before histopathologic review), PDTC, n = 21 (18%); and benign primary thyroid tumors (eg, adenomatous nodules or follicular adenomas), n = 2 (2%). Those 2 patients with benign primary thyroid tumors had distant metastasis even though malignant findings were not observed in the primary thyroid tumor specimens [21]. The median percentage of STI components was 40% (range, 0%-10%) in all patients, 5% (range, 0%-50%) in MI-FTC, 30% (range, 0%-100%) in EA-FTC, 30% (range, 0%-100%) in WI-FTC, 28% (range, 5%-70%) in DHGTC, and 70% (range, 50%-100%) in PDTC.

**Table 1:** Baseline patient characteristics

	All (n = 114)	MI-FTC (n = 5)	EA-FTC (n = 41)	WI-FTC (n = 23)	DHGTC (n = 22)	PDTC (n = 21)
Sex						
Male	28 (25%)	2 (40%)	7 (17%)	6 (26%)	6 (27%)	7 (33%)
Female	86 (75%)	3 (60%)	34 (83%)	17 (74%)	16 (773%)	14 (67%)
Age at diagnosis (years), median (range)	59 (10-77)	52 (38-72)	56 (10-76)	58 (12-77)	61 (14-76)	65 (29-75)
Age at diagnosis (years)						
<55	42 (37%)	3 (60%)	16 (39%)	10 (43%)	7 (32%)	6 (29%)
≥55	72 (63%)	2 (40%)	25 (61%)	13 (57%)	15 (68%)	15 (71%)
Age at first RAIT (years), median (range)	61 (11-78)	54 (38-73)	60 (11-77)	59 (13-77)	65 (16-78)	67 (33-75)
Age at first RAIT (years)						
<55	35 (31%)	3 (60%)	11 (27%)	10 (44%)	6 (27%)	5 (24%)
≥55	79 (69%)	2 (40%)	30 (73%)	13 (56%)	16 (73%)	16 (76%)
Tumor size (mm), median	46 (10-123)	50 (10-100)	45 (12-90)	39 (14-123)	61 (23-86)	60 (22-120)
Tumor size (mm)						
≤40	44 (39%)	2 (40%)	16 (39%)	12 (52%)	7 (32%)	7 (33%)
>40	70 (61%)	3 (60%)	25 (61%)	11 (48%)	15 (68%)	14 (67%)
T classification						
T1/T2	39 (34%)	2 (40%)	15 (37%)	11 (48%)	6 (27%)	5 (24%)
T3/T4	75 (66%)	3 (60%)	26 (63%)	12 (52%)	16 (73%)	16 (76%)
N classification						
N0/NX	106 (93%)	5 (100%)	39 (95%)	20 (87%)	20 (91%)	20 (95%)
N1	8 (7%)	0	2 (5%)	3 (13%)	2 (9%)	1 (5%)
M classification						
M1	70 (61%)	2 (40%)	22 (54%)	19 (83%)	10 (45%)	16 (71%)
M2	44 (39%)	3 (60%)	19 (46%)	4 (17%)	12 (55%)	5 (29%)
STI components (%), median (range)	40 (0-100)	5 (0-50)	30 (0-100)	30 (0-100)	28 (5-70)	70 (50-100)
Tg level at diagnosis (ng/mL), median (range)	1747.0 (11.0-81644.0)	1860.0 (328.0-12861.0)	1774.0 (11.0-36454.0)	988.0 (57.9-15426.0)	2933.0 (281.0-81644.0)	4138.0 (34.9-47497.0)
Thyroglobulin antibody	36*^a^* (32%)	2 (40%)	12 (29%)	4 (17%)	8*^a^* (38%)	10 (48%)
Disease sites at first RAIT						
Lung	78 (68%)	1 (20%)	28 (68%)	13 (57%)	17 (77%)	17 (81%)
Bone	66 (58%)	3 (60%)	25 (61%)	14 (61%)	11 (50%)	11 (52%)
Lymph node	13 (11%)	2 (40%)	2 (5%)	2 (9%)	6 (27%)	1 (5%)
Others	3 (3%)	0	1 (2%)	0	1 (5%)	1 (5%)
Systemic drug therapy	33 (29%)	2 (40%)	11 (27%)	3 (13%)	10 (45%)	7 (33%)

Abbreviations: DHGTC, differentiated high-grade thyroid carcinoma; EA, encapsulated angio-invasive; FTC, follicular thyroid carcinoma; MI, minimally invasive; PDTC, poorly differentiated thyroid carcinoma; RAIT, radioactive iodine treatment; STI, solid/trabecular/insular; WI, widely invasive.

^*a*^Data was unknown in 1 patient, and the percentage was calculated excluding this 1 case.

### Details of RAIT

The details of RAIT are shown in Table 2. Of the 114 patients, the median dose of the first RAIT, total RAI dose, and number of RAIT were 3952 MBq (107 mCi) (range, 3637-5550 MBq [98-150 mCi]), 7469 MBq (202 mCi) (range, 3700–42 090 MBq [100-1089 mCi]), and 2 (range, 1-9), respectively. The median Tg level before the first RAIT and stimulated Tg level at the first RAIT were 82.9 ng/mL (range, 0.1-93359.0 ng/mL) and 508.2 ng/mL (0.2-355220.0 ng/mL), respectively, and a cutoff value of 80 ng/mL for Tg level before the first RAIT and 500 ng/mL for stimulated Tg level at the first RAIT [10] were used for subsequent analyses. A total of 42 (37%) of 114 patients achieved Tg level reduction of ≥50% after the first RAIT. Among 77 patients without TgAb, the median Tg level before the first RAIT and stimulated Tg level at the first RAIT were 50.8 ng/mL (range, 0.1-93359.0 ng/mL) and 250.6 ng/mL (0.5-355220.0 ng/mL), respectively. Twenty-nine (38%) of 77 patients achieved Tg level reduction of ≥50% after the first RAIT.

**Table 2:** Details of radioactive iodine treatment

	All (n = 114)	MI-FTC (n = 5)	EA-FTC (n = 41)	WI-FTC (n = 23)	DHGTC (n = 22)	PDTC (n = 21)
Administration dose of first RAIT (MBq), median (range)	3952 (3637-5550)	3732 (3700-3955)	3952 (3700-5550)	3952 (3732-4304)	3844 (3700-4076)	3952 (3726-4076)
Total RAI dose (MBq), median (range)	7469 (3700-42 090)	11 388 (3952-15 607)	7464 (3700-23 408)	7473 (3732-27 922)	4076 (3700-42 090)	8027 (3952-24 447)
Tg level before first RAIT (ng/mL), median (range)	82.9 (0.1-93359.0)	25.6 (0.1-1210.0)	71.7 (0.1-15989.0)	62.4 (0.7-9049.0)	428.5 (0.1-93359.0)	212.2 (0.7-5027.0)
Stimulated Tg level at first RAIT (ng/mL), median (range)	508.2 (0.5-355220.0)	78.8 (1.3-5365.0)	353.8 (0.5-61688.0)	250.6 (2.7-33266.0)	3278.5 (0.9-355220.0)	534.1 (1.5-18905.0)
Number of RAIT, median (range)	2 (1-9)	3 (1-4)	2 (1-6)	2 (1-7)	1 (1-9)	2 (1-6)
Tg level reduction of ≥ 50% after first RAIT	42 (37%)	1 (20%)	16 (39%)	12 (52%)	6 (27%)	7 (33%)

Abbreviations: DHGTC, differentiated high-grade thyroid carcinoma; EA, encapsulated angio-invasive; FTC, follicular thyroid carcinoma; MI, minimally invasive; PDTC, poorly differentiated thyroid carcinoma; RAIT, radioactive iodine treatment; Tg, thyroglobulin; WI, widely invasive.

### Histology and RAI avidity

The RAI avidity for each histological type is shown in Table 3. Among the 114 patients, the rate of RAI avidity was 60% (95% CI, 49%-71%) (47/78) in lung, 89% (95% CI, 79%-96%) (59/66) was in bone, 100% (13/13) in lymph node, and 100% (3/3) in other sites, respectively. The rate of RAI avidity of lung was 0% (0/1) in MI-FTC, 61% (95% CI, 41%-79%) (17/28) was in EA-FTC, 77% (95% CI, 46%-95%) (10/13) was in WI-FTC, 59% (95% CI, 33%-82%) (10/17) was in DHGTC, 53% (95% CI, 28%-77%) (9/17) was in PDTC, and 50% (95% CI, 1%-99%) (1/2) was in others, respectively. In addition, the rate of RAI avidity of bone was 100% (3/3) in MI-FTC, 92% (95% CI, 74%-99%) (23/25) was in EA-FTC, 71% (95% CI, 42%-92%) (10/14) was in WI-FTC, 91% (95% CI, 59%-82%) (10/11) was in DHGTC, 100% (11/11) was in PDTC, and 100% (95% CI, 1%-99%) (2/2) was in others, respectively.

**Table 3:** Histology and first radioactive iodine treatment avidity

	All	MI-FTC	EA-FTC	WI-FTC	DHGTC	PDTC	Others
Lung	60% (95% CI 49-71%) (47/78)	0% (0/1)	61% (95% CI 41-79%) (17/28)	77% (95% CI 46-95%) (10/13)	59% (95% CI 33-82%) (10/17)	53% (95% CI 28-77%) (9/17)	50% (95% CI 1-99%) (1/2)
Bone	89% (95% CI 79-96%) (59/66)	100% (3/3)	92% (95% CI 74-99%) (23/25)	71% (95% CI 42-92%) (10/14)	91% (95% CI 59-99%) (10/11)	100% (11/11)	100% (2/2)
Lymph node	100% (13/13)	100% (2/2)	100% (2/2)	100% (2/2)	100% (6/6)	100% (1/1)	—
Other sites	100% (3/3)	—	100% (1/1)	—	100% (1/1)	100% (1/1)	—

Abbreviations: DHGTC, differentiated high-grade thyroid carcinoma; EA, encapsulated angio-invasive; FTC, follicular thyroid carcinoma; MI, minimally invasive; PDTC, poorly differentiated thyroid carcinoma; WI, widely invasive.

### Analyses of predictive factors for first RAIT avidity

Among all 114 patients, 94 (82%) had RAI avidity at any disease site. In addition, 62 (81%) patients had RAI avidity at any disease site among 77 patients without TgAb. Among those 77 patients without TgAb, patients without RAI avidity at any disease site had a stimulated Tg level at first RAIT > 500 ng/mL less frequently (13%, 2/13) than patients with RAI avidity at any disease site (47%, 29/62) (*P* = .020).

#### (i) Lung metastasis

The characteristics of patients with and without RAI avidity for lung metastasis are shown in Table 4. Of the 78 patients with lung metastasis, 47 (60%) had RAI avidity of lung metastasis. No significant predictive factors were identified, including the percentage of STI components. The median percentage of STI components were 30% (range, 0%-100%) in patients with lung metastasis avidity, and 40% (range, 0%-100%) in patients without lung metastasis avidity, respectively (*P* = .207).

**Table 4:** Univariate analysis of predictive factor for first radioactive iodine treatment avidity of lung metastasis

	Lung metastasis (n = 78)		Univariate analysis
	Avidity (+) (n = 47)	Avidity (−) (n = 31)	*P* value
Sex			.170
Male	8 (17%)	10 (32%)	
Female	39 (83%)	21 (68%)	
Age at diagnosis (years)			.333
<55	14 (30%)	13 (42%)	
≥55	33 (70%)	18 (58%)	
Age at first RAIT (years)			.071
<55	9 (38%)	12 (39%)	
≥55	38 (62%)	19 (61%)	
Histology			.641
Non-HGFCTC	28 (60%)	16 (52%)	
HGFCTC	19 (40%)	15 (48%)	
Tumor size (mm)			.157
≤40	22 (47%)	9 (29%)	
>40	25 (53%)	22 (71%)	
T classification			.328
T1/T2	18 (38%)	8 (26%)	
T3/T4	29 (62%)	23 (74%)	
N classification			.677
N0/NX	44 (94%)	28 (90%)	
N1	3 (6%)	3 (10%)	
M classification			.161
M1	30 (64%)	14 (45%)	
M2	17 (36%)	17 (55%)	
STI components (%), median (range)	30 (0-100)	40 (0-100)	.207
Bone metastasis			1
Yes	22 (47%)	15 (48%)	
No	25 (53%)	16 (52%)	
Tg level at diagnosis (ng/mL)			.645
≤1800	26 (55%)	15 (48%)	
>1800	21 (45%)	16 (52%)	
Tg level before first RAIT (ng/mL)			.356
≤80	20 (43%)	17 (55%)	
>80	27 (57%)	14 (45%)	
Stimulated Tg level at first RAIT (ng/mL)			.644
≤500	22 (47%)	17 (55%)	
>500	25 (53%)	14 (45%)	

Abbreviations: HGFCTC, high-grade non-anaplastic follicular cell–derived carcinoma; RAIT, radioactive iodine treatment; STI, solid/trabecular/insular; Tg, thyroglobulin.

#### (ii) Bone metastasis

The characteristics of patients with and without RAI avidity for bone metastasis are shown in Table 5. Of the 66 patients with bone metastasis, 59 (89%) had RAI avidity of bone metastasis. The median percentage of STI components were 30% (range, 0%-100%) in patients with bone metastasis avidity, and 20% (range, 0%-100%) in patients without bone metastasis avidity, respectively (*P* = .276). Univariate analysis showed that Tg level before the first RAIT > 80 ng/mL (*P* = .003) and stimulated Tg level at first RAIT > 500 ng/mL (*P* = .013) were significant predictive factors for RAI avidity of bone metastasis.

**Table 5:** Univariate analysis of predictive factor for first radioactive iodine treatment avidity of bone metastasis

	Bone metastasis (n = 66)		Univariate analysis
	Avidity (+) (n = 59)	Avidity (−) (n = 7)	*P* value
Sex			1
Male	14 (24%)	1 (14%)	
Female	45 (76%)	6 (86%)	
Age at diagnosis (years)			.687
<55	20 (34%)	3 (43%)	
≥55	39 (66%)	4 (57%)	
Age at first RAIT (years)			.425
<55	17 (29%)	3 (43%)	
≥55	42 (71%)	4 (57%)	
Histology			.409
Non-HGFCTC	38 (64%)	6 (86%)	
HGFCTC	21 (36%)	1 (14%)	
Tumor size (mm)			.690
≤40	25 (42%)	4 (57%)	
>40	34 (58%)	3 (43%)	
T classification			.420
T1/T2	22 (37%)	4 (57%)	
T3/T4	37 (63%)	3 (43%)	
N classification			.053
N0/NX	57 (97%)	5 (71%)	
N1	2 (3%)	2 (29%)	
M classification			.666
M1	40 (68%)	6 (86%)	
M2	19 (32%)	1 (14%)	
STI components (%), median (range)	30 (0-100)	20 (0-100)	.276
Lung metastasis			.690
Yes	34 (58%)	3 (43%)	
No	25 (42%)	4 (57%)	
Tg level at diagnosis (ng/mL)			.051
≤ 1800	26 (44%)	6 (86%)	
> 1800	33 (56%)	1 (14%)	
Tg level before first RAIT (ng/mL)			.003
≤80	23 (39%)	7 (100%)	
>80	36 (61%)	0	
Stimulated Tg level at first RAIT (ng/mL)			.013
≤500	20 (34%)	6 (86%)	
>500	39 (66%)	1 (14%)	

Abbreviations: HGFCTC, high-grade non-anaplastic follicular cell–derived carcinoma; RAIT, radioactive iodine treatment; STI, solid/trabecular/insular; Tg, thyroglobulin.

### Analysis of predictive factors for Tg level reduction after first RAIT

The characteristics of patients with and without a Tg level reduction of ≥50% after the first RAIT are shown in Table 6. Of the 114 patients, 42 (37%) had a Tg level reduction of ≥50% after the first RAIT. Among those 42 patients with a Tg level reduction of ≥50% after the first RAIT, 12 patients (29%) had TgAb. Univariate analysis showed that patients with Tg level reduction of ≥50% after the first RAIT had significantly more frequent M1 disease (*P* = .047) and lower STI components (*P* = .048) (both of these *P* values were marginal) and Tg level before the first RAIT (*P* = .034). Predictive factors were also analyzed among 77 patients without TgAb, and no significant predictive factors were identified (Table 7). The analysis for patients with FTC is shown in Table S1 [22], and patients with HGFCTC is shown in Table S2 [22], respectively.

**Table 6:** Univariate analysis of predictive factor for thyroglobulin level reduction after first radioactive iodine treatment

	Tg level reduction		Univariate analysis
	≥ 50% (n = 42)	< 50% (n = 72)	*P* value
Sex			.177
Male	7 (17%)	21 (29%)	
Female	35 (83%)	51 (71%)	
Age at diagnosis (years)			.843
<55	16 (38%)	26 (36%)	
≥55	26 (62%)	46 (64%)	
Age at first RAIT (years)			.529
<55	11 (26%)	24 (33%)	
≥55	31 (74%)	48 (67%)	
Histology			.318
Non-HGFCTC	29 (69%)	42 (58%)	
HGFCTC	13 (31%)	30 (42%)	
Tumor size (mm)			.073
≤40	21 (50%)	23 (32%)	
>40	21 (50%)	49 (68%)	
T classification			.311
T1/T2	17 (40%)	22 (31%)	
T3/T4	25 (60%)	50 (69%)	
N classification			1
N0/NX	39 (93%)	67 (93%)	
N1	3 (7%)	5 (7%)	
M classification			.047
M1	31 (74%)	39 (54%)	
M2	11 (26%)	33 (46%)	
STI components (%), median (range)	20 (0-80)	40 (0-100)	.048
Lung metastasis			.679
Yes	30 (71%)	48 (67%)	
No	12 (29%)	24 (33%)	
Bone metastasis			.846
Yes	25 (60%)	41 (55%)	
No	17 (40%)	31 (43%)	
Tg level at diagnosis (ng/mL)			.438
≤1800	19 (45%)	39 (54%)	
>1800	23 (55%)	33 (46%)	
Tg level before first RAIT (ng/mL)			.034
≤80	15 (36%)	41 (57%)	
>80	27 (64%)	31 (43%)	
Stimulated Tg level at first RAIT (ng/mL)			.561
≤500	19 (45%)	38 (53%)	
>500	23 (55%)	34 (47%)	
RAI avidity at any disease site			.309
Yes	37 (88%)	57 (79%)	
No	5 (12%)	15 (21%)	

Abbreviations: HGFCTC, high-grade non-anaplastic follicular cell–derived carcinoma; RAIT, radioactive iodine treatment; STI, solid/trabecular/insular; Tg, thyroglobulin.

**Table 7:** Univariate analysis of predictive factor for thyroglobulin level reduction after first radioactive iodine treatment in 77 patients without thyroglobulin antibody

	Tg level reduction		Univariate analysis
	≥50% (n = 29)	<50% (n = 48)	*P* value
Sex			.592
Male	6 (21%)	14 (29%)	
Female	23 (79%)	34 (71%)	
Age at diagnosis (years)			.341
<55	13 (45%)	16 (33%)	
≥55	16 (55%)	32 (67%)	
Age at first RAIT (years)			.800
<55	8 (28%)	16 (33%)	
≥55	21 (72%)	32 (67%)	
Histology			.325
Non-HGFCTC	22 (76%)	31 (65	
HGFCTC	7 (24	17 (35)	
Tumor size (mm)			.159
≤40	16 (55%)	18 (37%)	
>40	13 (45%)	30 (63%)	
T classification			.474
T1/T2	13 (45%)	17 (35%)	
T3/T4	16 (55%)	31 (65%)	
N classification			.359
N0/NX	26 (90%)	46 (96%)	
N1	3 (10%)	2 (4%)	
M classification			.478
M1	19 (65%)	27 (56%)	
M2	10 (35%)	21 (44%)	
STI components (%), median (range)	40 (5-80)	40 (0-100)	.537
Lung metastasis			.216
Yes	22 (76%)	29 (60%)	
No	7 (24%)	19 (40%)	
Bone metastasis			.475
Yes	15 (52%)	30 (63%)	
No	14 (48%)	18 (37%)	
Tg level at diagnosis (ng/mL)			.461
≤1800	17 (59%)	33 (69%)	
>1800	12 (41%)	15 (31%)	
Tg level before first RAIT (ng/mL)			.151
≤80	14 (48%)	32 (67%)	
>80	15 (52%)	16 (33%)	
Stimulated Tg level at first RAIT (ng/mL)			.633
≤500	16 (55%)	30 (63%)	
>500	13 (45%)	18 (37%)	
RAI avidity at any disease site			.774
Yes	24 (83%)	38 (79%)	
No	5 (17%)	10 (21%)	

Abbreviations: HGFCTC, high-grade non-anaplastic follicular cell–derived carcinoma; RAIT, radioactive iodine treatment; STI, solid/trabecular/insular; Tg, thyroglobulin.

### Clinical outcomes

During study period, the number of events of cause-specific death was 13. Among these 13 patients, 4 patients died before 2013. It is considered that 4 patients did not have chance to undergo systemic drug therapy for RAI-refractory progressive disease because systemic drug therapies such as sorafenib and lenvatinb were approved in 2014 and 2015, respectively, in Japan.

#### (i) Cause-specific survival from the diagnosis

The 10-year CSS-D rates of the patients with MI-FTC, EA-FTC, WI-FTC, DHGTC, PDTC, and others were 100%, 88.3%, 100%, 80.5%, 80.6%, and 50%, respectively. Univariate and multivariate analyses for CSS-D are shown in Table 8. The 10-year CSS-D rates of patients with percentage of STI < 40% and ≥40% were 90.6% and 83.6%, respectively (*P* = .794). Univariate analysis identified HGFCTC histology (*P* = .036) and Tg level before the first RAIT (*P* = .008) as prognostic factors for CSS-D. Among 56 patients with Tg level ≤80 ng/mL before the first RAIT, 10 patients (18%) had TgAb.

**Table 8:** Univariate analysis for cause-specific survival from the diagnosis

	n = 114	Univariate analysis	10-year CSS-D rate (%)
		*P* value	
Sex		.394	
Male	28 (25%)		96.4
Female	86 (75%)		83.5
Age at diagnosis (years)		.175	
<55	42 (37%)		90.7
≥55	72 (63%)		84.2
Age at first RAIT (years)		.592	
<55	35 (31%)		88.3
≥55	79 (69%)		86.1
Histology		.036	
Non-HGFCTC	71 (62%)		90.7
HGFCTC	43 (38%)		80.7
STI components (%)		.794	
<40	56 (49%)		90.6
≥40	58 (51%)		83.6
Tumor size (mm)		.715	
≤40	44 (39%)		89.1
>40	70 (61%)		85.7
T classification		.971	
T1/T2	39 (34%)		87.0
T3/T4	75 (66%)		86.8
N classification		.631	
N0/NX	106 (93%)		87.6
N1	8 (7%)		75.0
M classification		.090	
M1	70 (61%)		79.1
M2	44 (39%)		94.7
Lung metastasis		.724	
Yes	78 (67%)		85.5
No	36 (33%)		91.5
Bone metastasis		.976	
Yes	66 (58%)		85.0
No	58 (42%)		88.3
Tg level at diagnosis (ng/mL)		.349	
≤1800	58 (51%)		88.8
>1800	56 (49%)		84.5
Tg level before first RAIT (ng/mL)		.008	
≤80	56 (49%)		95.4
>80	58 (51%)		77.1
Stimulated Tg level at first RAIT (ng/mL)		.103	
≤500	57 (50%)		92.2
>500	57 (50%)		81.2
RAI avidity at any disease site		.448	
Yes	94 (82%)		88.3
No	20 (18%)		79.6
Number of RAIT		.622	
1	49 (43%)		87.6
≥2	65 (57%)		86.0
Tg level reduction after first RAIT		.626	
<50%	72 (63%)		86.3
≥50%	42 (37%)		88.4
Systemic drug therapy		.371	
Yes	33 (29%)		82.5
No	81 (71%)		88.7

Abbreviations: CSS-D, cause-specific survival from diagnosis; RAIT, radioactive iodine treatment; STI, solid/trabecular/insular; Tg, thyroglobulin.

The analysis for patients with FTC is shown in Table S3 [22], and patients with HGFCTC is shown in Table S4 [22], respectively.

#### (ii) Cause-specific survival from the first RAIT

The 10-year CSS-R rates of the patients with MI-FTC, EA-FTC, WI-FTC, DHGTC, PDTC, and other types were 100%, 80.3%, 100%, 49.9%, 72.2%, and not available, respectively. Univariate and multivariate analyses for CSS-R are shown in Table 9. The 10-year CSS-R rates of patients with percentage of STI < 40% and ≥40% were 78.3% and 75.1%, respectively (*P* = .563). Univariate analysis identified Tg level before the first RAIT (*P* = .004) as the only prognostic factor for CSS-R. The analysis for patients with FTC is shown in Table S5 [22], and patients with HGFCTC is shown in Table S6 [22], respectively.

**Table 9:** Univariate analysis for cause-specific survival from the first radioactive iodine treatment

	n = 114	Univariate analysis	10-year CSS-R rate (%)
		*P* value	
Sex		.438	
Male	28 (25%)		87.8
Female	86 (75%)		71.3
Age at diagnosis (years)		.216	
<55	42 (37%)		88.1
≥55	72 (63%)		68.0
Age at first RAIT (years)		.349	
<55	35 (31%)		86.4
≥55	79 (69%)		69.5
Histology		.060	
Non-HGFCTC	71 (62%)		88.0
HGFCTC	43 (38%)		58.3
STI components (%)		.563	
<40	56 (49%)		78.3
≥40	58 (51%)		75.1
Tumor size (mm)		.438	
≤40	44 (39%)		86.3
>40	70 (61%)		70.2
T classification		.639	
T1/T2	39 (34%)		86.3
T3/T4	75 (66%)		71.8
N classification		.918	
N0/NX	106 (93%)		75.6
N1	8 (7%)		75.0
M classification		.955	
M1	70 (61%)		77.2
M2	44 (39%)		75.3
Lung metastasis		.222	
Yes	78 (67%)		67.7
No	36 (33%)		91.1
Bone metastasis		.664	
Yes	66 (58%)		78.9
No	58 (42%)		74.0
Tg level at diagnosis (ng/mL)		.427	
≤1800	58 (51%)		86.7
>1800	56 (49%)		67.2
Tg level before first RAIT (ng/mL)		.004	
≤80	56 (49%)		93.8
>80	58 (51%)		51.7
Stimulated Tg level at first RAIT (ng/mL)		.079	
≤500	57 (50%)		88.4
>500	57 (50%)		61.3
RAI avidity at any disease site		.392	
Yes	94 (82%)		75.9
No	20 (18%)		71.4
Number of RAIT		.296	
1	49 (43%)		67.4
≥2	65 (57%)		81.8
Tg level reduction after first RAIT		.707	
<50%	72 (63%)		74.1
≥50%	42 (37%)		82.7
Systemic drug therapy		.683	
Yes	33 (29%)		71.4
No	81 (71%)		76.9

Abbreviations: CSS-R, cause-specific survival from first RAIT; RAIT, radioactive iodine treatment; STI, solid/trabecular/insular; Tg, thyroglobulin.

## Discussion

Clinicopathological risk factors, such as the postoperative histopathological type, are reportedly linked to a poor prognosis in FTC [16]. A recent study by Stegenga et al investigated the association between clinical outcomes and the fifth WHO classification of FTC and oncocytic carcinoma [23]. The 10-year disease-specific survival rates of MI-FTC (n = 32), EA-FTC (n = 34), and WI-FTC (n = 23) were 100%, 93.3%, and 85%, respectively, and were appropriately stratified [23]. A previous study that investigated RAI avidity and outcomes did not refer to the FTC subclassification [8, 10]. On the other hand, a large number of studies on clinical outcomes and RAI therapy among FTC patients using the Surveillance, Epidemiology, and End Results database, reported FTC subclassifications, and RAI therapy was found to have an advantage, in terms of CSS, in patients with WI-FTC who had N1 disease or distant metastasis at the diagnosis [24]. However, this study assessed only the presence or absence of RAI therapy [24]. Although the number of patients in our study was relatively small, this study has the strength that it provides detailed information, including FTC subclassification, metastatic disease sites, and the RAI avidity of individual disease sites.

DHGTC is a newly defined entity in the fifth WHO classification, and the concept was introduced by studies on the impact of nuclear atypia, tumor necrosis, vascular invasion, and mitotic rate on survival [25]. Xu et al investigated the clinicopathological factors among 364 patients with primary high-grade non-anaplastic thyroid carcinoma (HGTC), among whom 200 patients (54.9%) were diagnosed with PDTC based on the Turin consensus (HGTC-PDTC) and 164 with high-grade features that did not meet the Turin criteria (HGTC-nonPDTC) [26]. Among the 220 of 364 patients with HGTC, 116 (52.7%) had RAI avidity, which was associated with HGTC-PDTC histology (RAI-avid: 65%, not RAI-avid: 51%, *P* = .042) [26]. Then, 41 HGTC-nonPDTCs with RAI avidity consisted of 19 follicular variant PTCs (46%), 7 tall cell PTCs (17%), 7 classic PTCs (17%), 3 diffuse sclerosing variant PTCs (7%), and 5 FTCs (12%) [26]. Although the number of FTCs was very small (6 of 220 HGTCs), 5 of 6 FTC patients had RAI avidity [26]. In our study, RAI avidity of the lungs and bones was detected in 63% and 91% of DHGTCs and 53% and 100% of PDTCs, respectively. The results of our study and the study of Xu et al differed in point of the analysis of RAI avidity after the initial surgery and RAI avidity for individual distant metastatic sites. Therefore, these results could not be evaluated as the same RAI avidity. However, RAI avidity may be relatively favorable in patients with DHGTC if the tumors are derived from FTC.

Simões-Pereira et al showed that RAI avidity was highest in follicular variant PTC (75.6%) and FTC (76.5%) among 126 patients, including classical variant PTC (n = 42), follicular variant PTC (n = 45), FTC (n = 17), and Hürthle cell carcinoma (n = 22), and histology showed an independent association with RAI avidity [10]. In our study, 94 of 114 (82%) patients showed any RAI avidity, and this high RAI avidity rate was in line with previous reports [10, 26]. Considering that RAI avidity was higher in follicular variant PTC and FTC, it is speculated that follicular structure is associated with RAI avidity. However, no significant association was observed between the percentage of STI components in primary thyroid tumors and RAI avidity in both lung and bone metastases. Therefore, other factors such as genetic mutations [13, 27], may affect RAI avidity, and further studies are needed to identify useful predictive factors for RAI avidity among FTC, DHGTC, and PDTC.

This study had some limitations. First, it was a retrospective cohort study that included a small number of patients. Second, we analyzed only RAI avidity in each metastatic disease site and did not consider the intensity of RAI uptake in post-RAIT WBS, which probably had an impact on Tg level reduction and the patient prognosis. Third, the size of the metastatic disease site was not considered in this analysis. In particular, it was reported that the size of lung metastases is associated with RAI avidity [28]. However, some bone metastases cannot be identified by imaging and only have RAI avidity. In these cases, accurate measurement of size is often difficult. Thus, Tg level may be an alternative marker for monitoring changes in tumor burden. Fourth, there was the heterogeneity of treatment exposure such as total RAI dose or administration of systemic therapy that probably affected the analyses in CSS. Fifth, molecular data was not available in this cohort because molecular testing is not routinely performed before RAI treatment in Japan. Finally, a pathological examination of the distant metastatic site was not performed. It is possible that the histology and pathological morphology were different between primary thyroid tumors and metastatic disease lesions. In fact, a different histology between the primary tumor and metastasis was observed in 16 (37%) of 43 patients [29]. In addition, 10 (63%) of the 16 patients showed transformation to a higher grade (eg, transformation to PDTC from a follicular variant PTC) [29]. If follicular structure is associated with RAI avidity in thyroid cancer, the heterogeneity between primary thyroid tumor and distant metastasis may affect this study results. However, in the management of thyroid carcinoma, biopsies of distant metastatic sites are not always performed when distant metastases appear, and the histology of the primary thyroid tumor is considered for the induction of RAI therapy. Given that RAI avidity was relatively preserved even in patients with DHGTC and PDTC in our study, concern about transformation to higher-grade disease in distant metastases may be unnecessary when considering the first RAIT in non-papillary follicular cell-derived carcinomas.

In conclusion, RAI avidity was relatively high in non-papillary follicular cell–derived thyroid carcinoma, regardless of the histological type and percentage of STI components. Further research, including a genetic mutation analysis, is needed to clarify the relationship between histological findings and RAI treatment in patients with non-papillary follicular cell–derived thyroid carcinoma.

## Data Availability

Some or all datasets generated during and/or analyzed during the current study are not publicly available but are available from the corresponding author on reasonable request.

## Abbreviations

CSS-D: cause-specific survival from the diagnosis
CSS-R: cause-specific survival from radioactive iodine treatment
DHGTC: differentiated high-grade thyroid carcinoma
DTC: differentiated thyroid carcinoma
EA-FTC: encapsulated angio-invasive follicular thyroid carcinoma
FTC: follicular thyroid carcinoma
HGFCTC: high-grade non-anaplastic follicular cell–derived carcinoma
HGTC: high-grade non-anaplastic thyroid carcinoma
MI-FTC: minimally invasive follicular thyroid carcinoma
PDTC: poorly differentiated thyroid carcinoma
PTC: papillary thyroid carcinoma
RAI: radioactive iodine
RAIT: radioactive iodine treatment
STI: solid/trabecular/insular
Tg: thyroglobulin
WBS: whole-body I-131 scintigraphy
WHO: World Health Organization
WI-FTC: widely invasive follicular thyroid carcinoma
